# In vitro and in vivo effects of 3-bromopyruvate against *Echinococcus* metacestodes

**DOI:** 10.1186/s13567-019-0710-7

**Published:** 2019-11-19

**Authors:** Qi Xin, Miaomiao Yuan, Huanping Li, Xiaoxia Song, Jun Lu, Tao Jing

**Affiliations:** 1 Institute of Pathogenic Biology, School of Basic Medical Sciences, Lanzhou University, Lanzhou, China; 2 The Eighth Affiliated Hospital, Sun Yat-sen University, Shenzhen, China

## Abstract

While searching for novel anti-echinococcosis drugs, we have been focusing on glycolysis which is relied on by *Echinococcus* for energy production and intermediates for other metabolic processes. The aim of this study was to investigate the potential therapeutic implication of glycolytic inhibitors on *Echinococcus*. Our results demonstrate that at an initial concentration of 40 μM, all inhibitors of glycolysis used in the current experiment [3-bromopyruvate (3-BrPA), ornidazole, clorsulon (CLS), sodium oxamate and 2,6-dihydroxynaphthalene (NA-P_2_)] show considerable in vitro effects against *Echinococcus granulosus* protoscoleces and *Echinococcus multilocularis* metacestodes. Among them, 3-BrPA exhibited the highest activity which was similar to that of nitazoxanide (NTZ) and more efficacious than albendazole (ABZ). The activity of 3-BrPA was dose dependent and resulted in severe ultrastructural destructions, as visualized by electron microscopy. An additional in vivo study in mice infected with *E. multilocularis* metacestodes indicates a reduction in parasite weight after the twice-weekly treatment of 25 mg/kg 3-BrPA for 6 weeks, compared to that of the untreated control. In particular, in contrast to ABZ, the administration of 25 mg/kg 3-BrPA did not cause toxicity to the liver and kidney in mice. Similarly, at the effective dose against *Echinococcus* larvae, 3-BrPA showed no significant toxicity to human hepatocytes. Taken together, the results suggest that interfering with the glycolysis of the parasite may be a novel chemotherapeutical option and 3-BrPA, which exhibited a remarkable activity against *Echinococcus*, may be a promising potential drug against cystic echinococcosis (CE) and alveolar echinococcosis (AE).

## Introduction

Echinococcosis, caused by the larval stage of the parasitic cestode *Echinococcus* is a serious but neglected helminthic zoonosis. Cystic echinococcosis (CE) caused by *Echinococcus granulosus* is endemic in regions of western and central Europe, eastern Europe, North America and Asia, particularly in China [1], where CE is endemic in at least 23 provinces, with an estimated 380,000 patients and nearly 66 million individuals at risk of infection [2, 3]. Alveolar echinococcosis (AE) caused by *Echinococcus multilocularis* is endemic in the Northern hemisphere, and the greatest prevalence is found in Central Asia, Russia, north-western China, parts of Europe and Japan [4]. A global estimate of AE suggests that approximately 18,000 (CIs 11,932–28,156) new cases every year and a total of 0.3–0.5 million AE cases are diagnosed. Moreover, most of the disease (over 90%) of AE occurs in western China [5]. Both diseases are acquired through the accidental ingestion of parasite eggs shed by the definitive hosts (mainly dogs for *E. granulosus* and foxes and dogs for *E. multilocularis*). Upon infection, the larval stage of *Echinococcus* proliferate asexually in humans or other intermediate hosts, leading to space-occupying lesions, organ malfunction and even death [6].

Currently, percutaneous treatment, surgery and anti-infective drug treatment are the treatment options for CE. Radical surgical resection of the parasitic mass is the basis of treatment for AE and accompanied usually by chemotherapy. However, in the inoperable and the recrudescent patients, chemotherapy is the only option [7]. Benzimidazole derivatives such as albendazole (ABZ) and mebendazole are the only two drugs licensed for the treatment of human CE and AE [8]. However, the cure rate of these drugs against CE was reported to be only about 30% and besides, 20–40% of patients do not respond favorably. For treating AE, these drugs exhibit a parasitostatic rather than parasitocidal effect [9] and therefore, the patients have to undergo lifelong application of these drugs, which can cause hepatotoxicity. In addition, adverse side effects have been observed with these drugs [10, 11]. Thus, the development of novel therapeutic drugs for human CE and AE is urgently needed. Furthermore, novel parasitocidal drugs might be valuable and promising strategies for the control programs of CE, which may reduce the viability of protoscoleces in infected ungulate hosts and therefore reduce the infection of dogs.

In the organs of the intermediate host, the parasite, especially *E. multilocularis* metacestodes, exhibit properties of asexual, unlimited and infiltrative proliferation which is similar to malignant tumors. Hence, a number of antitumor drugs have been investigated in vitro and in vivo and have exhibited promising effects against *Echinococcus* metacestodes and protoscoleces, such as 2-methoxyestradiol [12], imatinib [13], doxorubicin [14], cyclosporine [15], isoflavone genistein and the genistein derivative [16], artemisinin and artemisinin derivatives [17], tamoxifen [18], bortezomib [19], 5-fluorouracil and paclitaxel [20, 21]. Like other parasites, the larval stage of *Echinococcus* obtains glucose from their hosts as their energy source. Although *E. granulosus* and *E. multilocularis* protoscoleces both have aerobic and anaerobic respiratory systems, there are no significant differences observed in the rate of glycogen utilization under aerobic or anaerobic conditions, which indicates that they both depend on glycolysis for energy and intermediates production [22, 23]. So, the targeting of anaerobic glycolysis in *Echinococcus* and inhibiting the glycolytic enzymes, such as hexokinase (HK), glyceraldehyde 3-phosphate dehydrogenase (GAPDH), fructose 1,6-bisphosphate aldolase (ALDO), phosphoglycerate kinase (PGK), phosphoglyceratmutase (PGM) and lactate dehydrogenase (LDH) may interfere with energy-yielding pathways, abolish ATP generation and finally eliminate the *Echinococcus* metacestodes. Several glycolytic inhibiting agents, such as 3-bromopyruvate (3-BrPA) [24–26], ornidazole [27], clorsulon (CLS) [28], sodium oxamate [29–31] and 2,6-dihydroxynaphthalene (NA-P_2_) [32], have been known to abolish ATP generation through glycolysis. Accordingly, in the present study, we used a defined experimental system to assess the potential therapeutic implications based on the metabolic signature of *Echinococcus* metacestodes by investigating the in vitro and in vivo effects of glycolytic inhibitors mentioned above against *E. granulosus* and *E. multilocularis*. Our results demonstrate the effective activity of glycolytic inhibitors against *E. granulosus* protoscoleces and *E. multilocularis* metacestodes and 3-BrPA will be a promising drug for the treatment of CE and AE.

## Materials and methods

### Chemicals

ABZ, NTZ, 3-BrPA, ornidazole, CLS, NA-P_2_, and sodium oxamate were obtained from Sigma-Aldrich (St. Louis, MO, USA). All tissue culture media and fetal calf serum (FCS) were purchased from Hyclone (Logan, UT, USA).

### Ethics statement

Animal procedures and management were carried out in accordance with the protocols (2014-12-003) approved by the Institutional Animal Caring and Using Committee of Lanzhou University. Unnecessary animal suffering was avoided throughout the study. The animals were housed in a temperature-controlled, light-cycled room. Food and water were given ad libitum.

### In vitro drug treatment of *E. granulosus* protoscoleces

*Echinococcus granulosus* protoscoleces were isolated aseptically from hydatid cysts in the liver of infected sheep slaughtered in an abattoir (Xining, Qinghai, China) and washed twice in Hanks balanced salt solution. The in vitro drug treatment of protoscoleces was performed as previously described [33]. One hundred viable and morphologically intact protoscoleces per well were cultured using RPMI 1640 culture medium without FCS and phenol red in 24-well tissue culture plates under 37 °C, 5% CO_2_ conditions. All drugs were prepared as stock solutions of 20 mM in dimethyl sulfoxide (DMSO) and added to the wells to a final concentration of 40 μM for initial screening. The drugs showing good efficacy were further investigated. For the dose-dependent effects of 3-BrPA, final concentrations of 10, 20, 40, 60, 80, and 100 μM were tested. Protoscoleces incubated in culture medium containing 0.2% DMSO were used as controls. The effect of drugs on the morphology and structural integrity of protoscoleces was observed microscopically every day for a period of 7 days and viability was assessed daily by trypan blue staining test. In short, after centrifuging at 10 000 × *g* for 30 s, the precipitated protoscoleces were mixed with 0.04% (w/v) trypan blue for 3 min. Finally, the mortality rate of the protoscoleces was determined under a microscope. Each drug concentration was performed in duplicate and each experiment was repeated twice.

### In vitro drug treatment of *E. multilocularis* metacestodes

In order to assess in vitro efficacy of the drugs against *E. multilocularis* metacestodes, an in vitro cultivation of *E. multilocularis* metacestodes (isolate Xinjiang) was first carried out as previously described with few modifications [34]. In short, metacestodes dissected from experimentally infected Mongolian gerbils (*Meriones unguiculatus*) were cut into small tissue blocks of 0.5 cm^3^ and washed twice in Hanks balanced salt solution. Three pieces of tissue were placed in a 25-cm^2^ cell culture flask containing human liver SMMC-7721 cells cultivation medium (Dulbecco modified Eagle medium [DMEM], supplemented with 12 mM HEPES, 2 mM glutamine, 100 U/mL penicillin, 100 μg/mL streptomycin and 10% FCS). These co-cultures were incubated at 37 °C, 5% CO_2_, with medium changes thrice a week. The metacestode vesicles were used for in vitro drug efficacy studies when they reached 2 to 4 mm in diameter. The metacestode vesicles harvested after 1–2 months of in vitro culture were washed three times in Hanks balanced salt solution and again distributed to 24-well plates (Corning Inc., New York, NY, USA) with approximate 30 vesicles per well. Treatments were carried out in 2 mL of RPMI 1640 culture medium without FCS and phenol red (2 mM glutamine, 100 U/mL penicillin, 100 μg/mL streptomycin), in which the drugs were added to a final concentration of 40 μM for initial screening. The drugs showing good efficacy were further investigated. For the dose-dependent effects of 3-BrPA, final concentrations of 5, 10, 20, 40, 80, and 100 μM were tested. Metacestodes incubated in culture medium containing 0.2% DMSO were used as controls. After 36 and 120 h of incubation, 300 μL of culture medium from all groups were collected respectively and centrifuged at 10 000 × *g* for 30 min at 4 °C. The supernatants were recovered and stored at −20 °C until measurements of *E. multilocularis* alkaline phosphatase (*Em*AP) activity.

### Determination of *Em*AP activity

The quantitative assessment of *Em*AP activity was carried out as previously described [35]. The assay was performed in 96-well microtiter plates (Corning Inc., New York, NY, USA) under axenic conditions. Per well, a 30 μL of supernatant aliquot was mixed with 170 μL alkaline phosphatase substrate buffer (0.5 M ethanolamine, 0.5 mM MgCl_2_ [pH 9.8]) containing *p*-nitrophenyl phosphate (1 mg/mL). The plates were incubated at 37 °C for 30 min. Then, *A*_405_ values were read on an ELISA (enzyme-linked immunosorbent assay) reader (Bio-Tek, Winooski, VT, USA). The measurements were performed in triplicate.

### Ultrastructural investigations of 3-BrPA treated metacestodes

At 120 h of treatment, the metacestodes were fixed and processed for scanning electron microscopy (SEM) and transmission electron microscopy (TEM). Briefly, the metacestodes were fixed in 2.5% glutaraldehyde in PBS (pH 7.2) for 24 h at 4 °C, followed by postfixation in 2% osmium tetraoxide (OsO_4_) in 100 mM PBS (pH 7.2) for 2 h at room temperature. For SEM analysis, the samples were washed in distilled water and dehydrated in various concentrations of ethanol (50, 70, 80, 90 and 100%) for 10 min at 4 °C. Subsequently, they were immersed in 2% isoamyl acetate, dried to a critical point and sputter-coated with gold. Finally, they were observed and photographed with a Hitachi S-450 SEM, with an acceleration voltage of 30 kV. For TEM analysis, the samples were washed in distilled water and treated with 1% uranyl acetate for 1 h. They were then rinsed extensively in distilled water, dehydrated in various concentrations of ethanol (50, 70, 80, 90 and 100%) and were embedded in Epon 812 resin. The embedded samples were dried by heat with serial temperatures (40 °C for 48 h and 60 °C for 48 h). Then they were cut into ultra-thin sections with a LKB-Nova ultramicrotome (LKB, Bromma, Sweden), mounted on a copper mesh grid, and stained with 3% uranyl acetate and lead citrate. Afterwards, the samples were observed at an acceleration voltage of 80 kV, in JEOL JEM-1230 TEM.

### Assessment of the efficacy of 3-BrPA treatment in experimentally infected BALB/c mice

3-BrPA was chosen for the in vivo assessment as it exhibited the best effect among selected glycolytic inhibitors in the in vitro experiment. Eight-week-old 18 to 20 g female BALB/c mice were injected intraperitoneally with 200 μL of homogenized *E. multilocularis* metacestode (isolate Xinjiang) tissue. The animals were separated into three groups of seven mice each and treatments were carried out as follows: group 1 received no drugs (200 μL honey/0.5% carboxymethyl cellulose (CMC) (1:1 v/v), untreated control group); group 2 received ABZ (200 mg/kg/day, ABZ control group); group 3 received 3-BrPA (25 mg/kg twice a week). The drugs were suspended in honey/0.5% CMC (1:1 v/v) and applied orally in a volume of 200 μL/mouse. The treatments began at 8 weeks post-infection and continued for 6 weeks. One week after the treatments stopped, the animals were sacrificed. After necropsy, the metacestode tissues were carefully isolated and recorded, and the parasite weight was determined for each animal.

### In vitro toxicity assessment of 3-BrPA on human hepatocytes

The human hepatocytes (7701 cell line) were purchased from the Cell Bank of Type Culture Collection Committee, Chinese Academy of Sciences, Shanghai. The cells were seeded into 96-well culture plates at a density of 10 000 cells/well and incubated in culture medium (RPMI 1640 containing 2 mM glutamine, 50 U/mL penicillin, 50 μg/mL streptomycin) for 24 h at 37 °C with 5% CO_2_. ABZ and 3-BrPA were added to the cultures at concentrations of 5, 10, 20, 40, 80, 100, 160 and 200 μM. As controls, the cells were performed with medium and corresponding amounts of DMSO present in the treated groups. After incubation for 48 h, the cell viability was examined by the MTT (3-(4,5-dimethylthiazol-2-yl)-2,5-diphenyltetrazolium bromide) reduction assay [36]. *A*_490_ values were read on an ELISA reader (Bio-Tek, Winooski, VT, USA) and the inhibition rate was calculated by this formula: [1 − (OD_treated_ − OD_blank_)/(OD_control_ − OD_blank_)] × 100%. The IC_50_ values were calculated in GraphPad Prism 5.

### In vivo toxicity assessment in mice

Twenty-one 6 to 8-week-old healthy female BALB/c mice were randomly divided into three groups and treatments were carried out as follows: group 1 received no drug treatment (200 μL honey/0.5% CMC (1:1 v/v), untreated control group); group 2 received ABZ (200 mg/kg/day); group 3 received 3-BrPA (25 mg/kg twice a week). The drugs were suspended in honey/0.5% CMC (1:1 v/v) and applied by intragastric inoculation in a volume of 200 μL/mouse. The treatments were performed for a period of 6 weeks. At the end of the study, whole blood via ocular sinus was collected from mice. Serum was separated by centrifugation (3000 × *g*, 10 min) following blood clotting. The serum levels of alanine aminotransferase (ALT), aspartate amino transferase (AST), total protein (TP), albumin (ALB), urea, creatinine (CREA), alkaline phosphatase (ALP), glutamyltranspeptidase (GGT), total bilirubin (TBIL) and direct bilirubin (DBIL) were determined with an automatic biochemical analyzer (Type 7170, HITACHI, Ltd., Tokyo, Japan). The mice were sacrificed to collect the tissues (liver and kidney). After fixation in 10% of neutral formaldehyde for 24 h, the tissues were embedded in paraffin. The samples were then sectioned (5 μm thickness) and stained with hematoxylin and eosin (HE) [37] for histopathological examination.

### Statistics analysis

Statistical analysis was done with SPSS 19.0 software. The results are presented as mean ± standard deviation (SD). One-way analysis of variance (ANOVA) was used to analyze the data on the effects of glycolytic inhibitors against *E. multilocularis* metacestodes. Nonparametric test of the Kruskal–Wallis test followed by Dunn multiple comparisons test were used for the assessment of efficacy of 3-BrPA treatment in the infected BALB/c mice and in vivo toxicity assessment in mice. The results are considered statistically significant for *p *< 0.05.

## Results

### In vitro effectivities of the selected glycolytic inhibitors against *E. granulosus* protoscoleces

**Figure 1:**
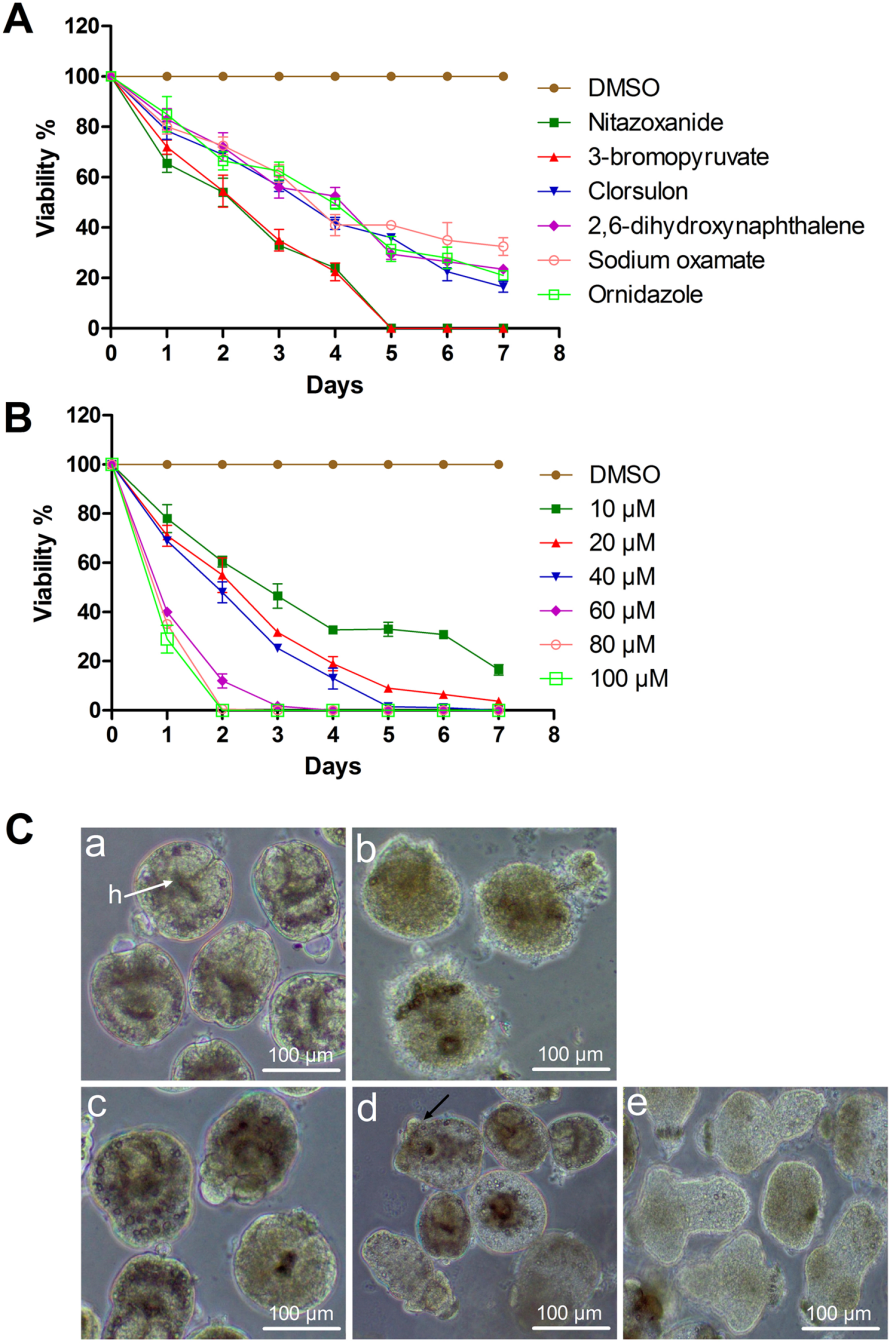
**In vitro effects of the selected glycolytic inhibitors against** ***E. granulosus*** **protoscoleces. A** Protoscoleces were incubated with each drug for up to 7 days, and the viability was measured by trypan blue exclusion test. All drugs were applied at a final concentration of 40 μM. **B** Concentration-dependent in vitro anti-protoscoleces effects by 3-BrPA, the most active drug, as a glycolytic inhibitor. Protoscoleces were incubated for 7 days with different concentrations (10 to 100 μM). **C** Light microscopy of protoscoleces incubated with 40 μM NTZ and 3-BrPA respectively. (Panel a) Protoscoleces incubated in culture medium containing DMSO served as a control. White arrow with h points towards hooks; (panel b) Protoscoleces incubated with NTZ for 5 days; (panel c) Protoscoleces incubated with 3-BrPA for 1 day; (panel d) Protoscoleces incubated with 3-BrPA for 3 days. Note the formation of blebs on the tegument (black arrow), collapse of suckers and decrease of calcium precipitates; (panel e) Protoscoleces incubated with 3-BrPA for 5 days. Note the protoscoleces were completely dead.

We investigated the in vitro effectivities of the selected glycolytic inhibitors on *E. granulosus* protoscoleces in comparison to that of NTZ, which served as a positive control, using an initial concentration of 40 μM and a treatment course of 7 days. Figure 1A shows the results of viability assays. The protoscoleces in DMSO control maintained 100% viability throughout the experimental period. After 7 days of culture, oxamate, NA-P_2_, ornidazole and CLS killed 65%, 76%, 79% and 85% of protoscoleces, respectively. Most notable of the drugs was 3-BrPA, which exhibited a similar profound activity against protoscoleces as NTZ, killing 100% of protoscoleces after only 5 days of treatment. Furthermore, 3-BrPA was subsequently evaluated in a concentration series (10, 20, 40, 60, 80 and 100 μM) for a period of 7 days (Figure 1B). The result demonstrates that 3-BrPA exerted a dose- and time-dependent effect against *E. granulosus* protoscoleces. At a concentration of 100, 80, 60 and 40 μM, 100% protoscoleces were killed within 2, 3, 4 and 5 days respectively. When tested at a concentration as low as 10 μM, 3-BrPA could still kill 67% of protoscoleces within 4 days and 84% of the protoscoleces within 7 days.

The results of the morphological observation by optical microscopy coincided with the viability assay as well. Figure 1C shows the morphological changes of the protoscoleces upon 40 μM 3-BrPA. After 1 day treatment, the posterior region of the protoscoleces became contracted (Figure 1C (panel c)); after 3 days, the protoscoleces exhibited extensive damages, such as collapse of suckers, formation of blebs on the tegument and decrease of calcium precipitates (Figure 1C (panel d)); after 5 days, the protoscoleces were completely dead with an apparent shrinkage of parenchyma (Figure 1C (panel e)). Severer morphological alterations were also seen after 5 days treatment with NTZ (Figure 1C (panel b)). The protoscoleces control maintained a normal morphology (Figure 1C (panel a)).

### In vitro effectivities of the selected glycolytic inhibitors against *E. multilocularis* metacestodes

**Figure 2:**
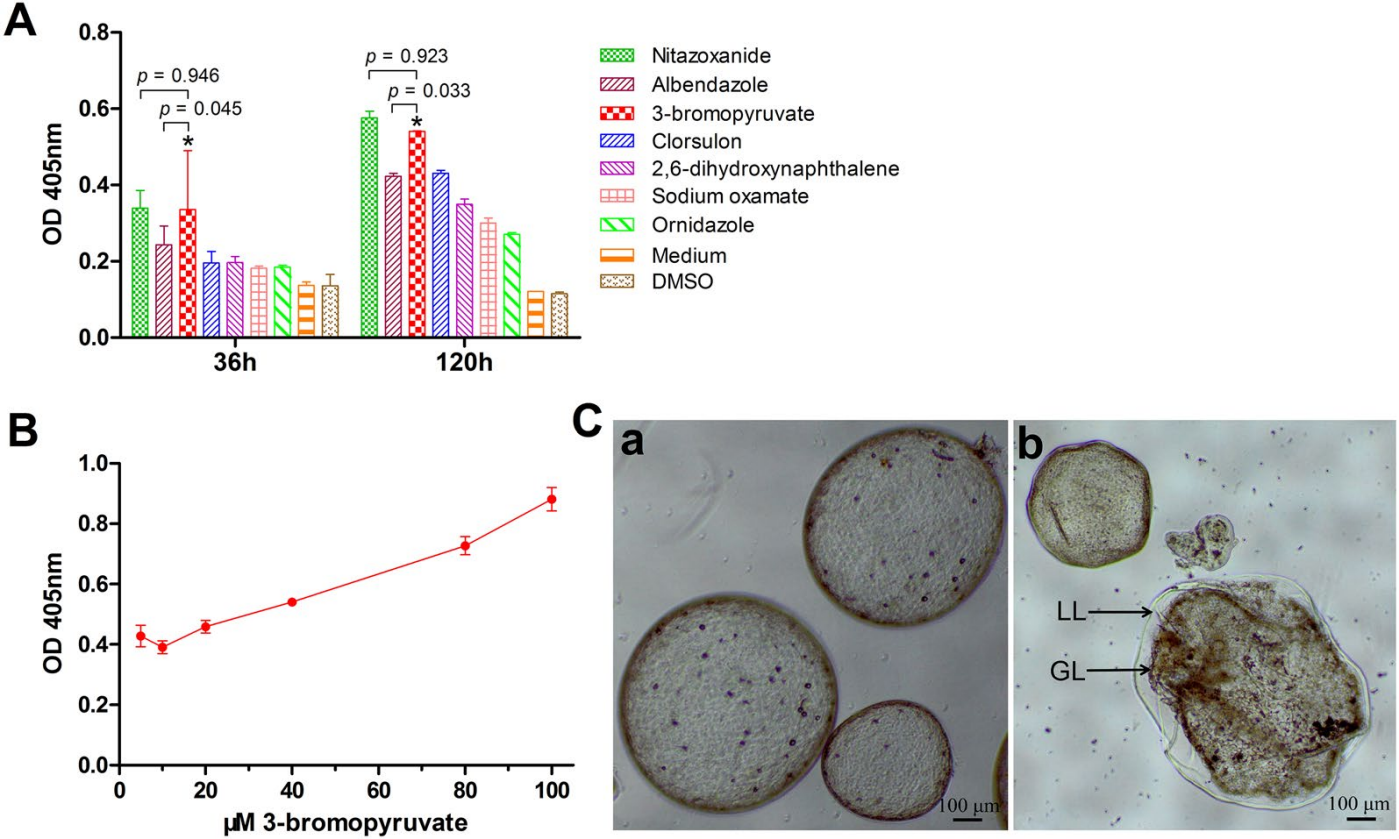
**In vitro effects of the selected glycolytic inhibitors against** ***E. multilocularis*** **metacestodes. A** Damage upon vesicles resulted from the selected drugs was assessed by measuring the *E. multilocularis* AP (*Em*AP) activities in the medium supernatant after 36 and 120 h of in vitro treatment. All drugs were added to a final concentration of 40 μM. Vesicles incubated in culture medium containing DMSO served as controls. *p < 0.05 vs. ornidazole, clorsulon, sodium oxamate, 2,6-dihydroxynaphthalene, medium and DMSO groups. **B** In vitro concentration-dependent *Em*AP activities after 120 h incubation with different concentrations (5 to 100 μM) of 3-BrPA. **C** Visualization of metacestodes after 36 h treatment of 40 μM 3-BrPA. (Panel a) Vesicles incubated in culture medium containing DMSO exhibit a normal morphological structure; (panel b) Metacestodes incubated in culture medium containing 40 μM 3-BrPA exhibit the loss of turgor, collapse of the vesicles, and detachment of the germinal layer from the laminated layer. LL: laminated layer, GL: germinal layer.

We investigated the *Em*AP activity in culture supernatants at 36 and 120 h following the addition of the selected glycolytic inhibitors into the culture medium. Figure 2A shows the results of *Em*AP assays carried out at an initial concentration of 40 μM. The results demonstrate that, in comparison to the DMSO control group, in vitro treatment with each of the selected glycolytic inhibitors could result in an increased release of AP activity, which indicates the damage to metacestodes. Obviously, the exposure for 120 h rendered a higher release of the *Em*AP than that for 36 h, which indicates that the effects of the glycolytic inhibitors were stable and time-dependent. Among the compounds, 3-BrPA resulted in 0.19 ± 0.05 and 0.44 ± 0.002 OD450 nm increase at 36 and 120 h of treatment respectively compared with the DMSO group, higher than the ABZ group (0.09 ± 0.009; 0.31 ± 0.002) and other groups except the NTZ group. These findings indicate that among these selected glycolytic inhibitors, 3-BrPA exhibited the most active effect against *E. multilocularis* metacestodes. In a subsequent experiment at 5, 10, 20, 40, 80 and 100 μM for a period of 120 h, it was demonstrated that the effect of 3-BrPA against *E. multilocularis* metacestodes was dose dependent (Figure 2B). In addition, when tested at 5 μM, 3-BrPA still showed a high anti-metacestode activity, with a 0.32 ± 0.001 OD450 nm increase compared with the DMSO group, which was also higher than the ABZ group (0.31 ± 0.002). The morphological alterations resulting from 3-BrPA were also visualized by light microscopy (Figure 2C). At 36 h of treatment, the vesicles exhibited loss of turgor, collapse of some vesicles, detachment of the germinal layer from the laminated layer, and formation of a densely packed aggregate inside the vesicles.

### Ultrastructural alterations of *E. multilocularis* metacestodes after 3-BrPA treatment

**Figure 3:**
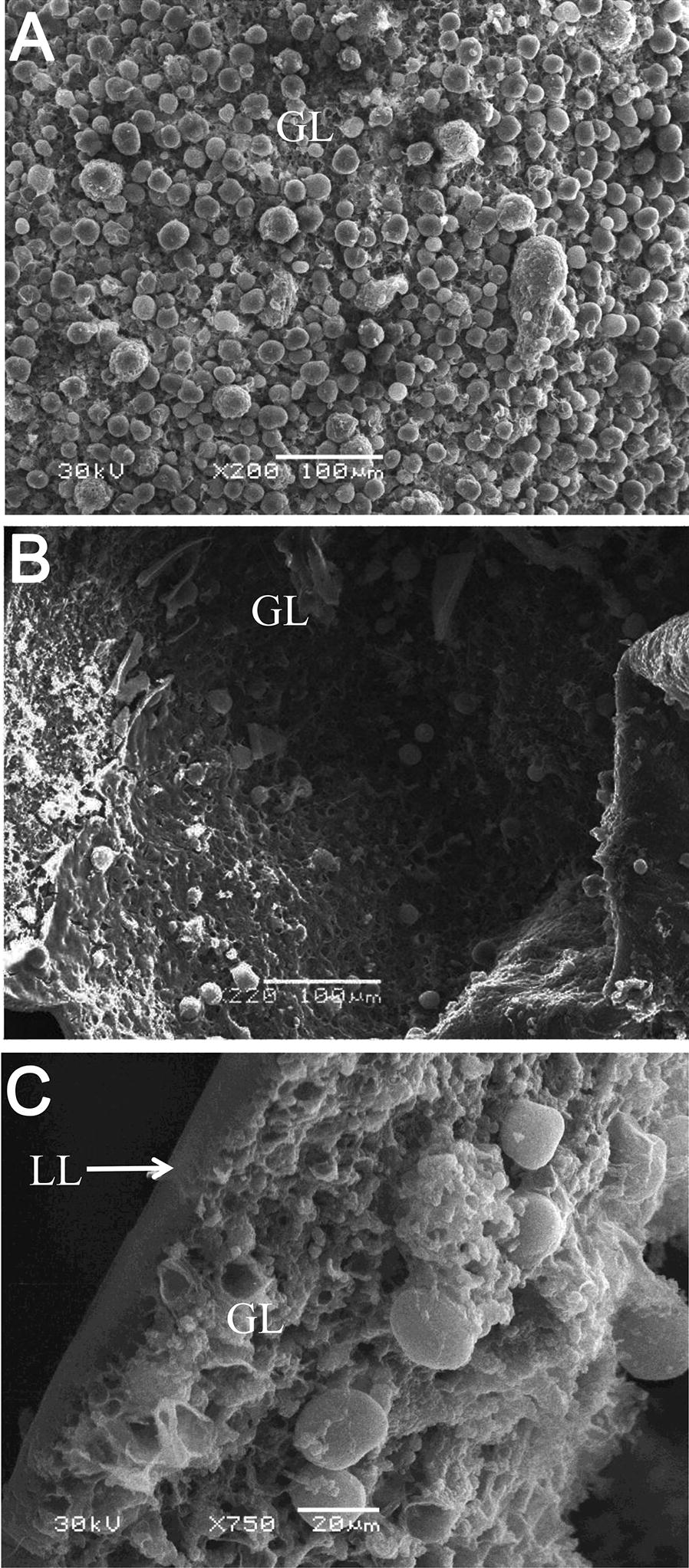
**SEM of** ***E. multilocularis*** **metacestodes incubated in vitro with 3-BrPA. A** Metacestodes incubated in culture medium containing DMSO served as controls. Note that the metacestode has a typical structure with an intact germinal layer. LL: laminated layer, GL: germinal layer. **B**, **C** The metacestodes incubated with 40 μM 3-BrPA for 120 h show the germinal layer detached from the laminated layer and the loss of cellular integrity of the germinal layer.

**Figure 4:**
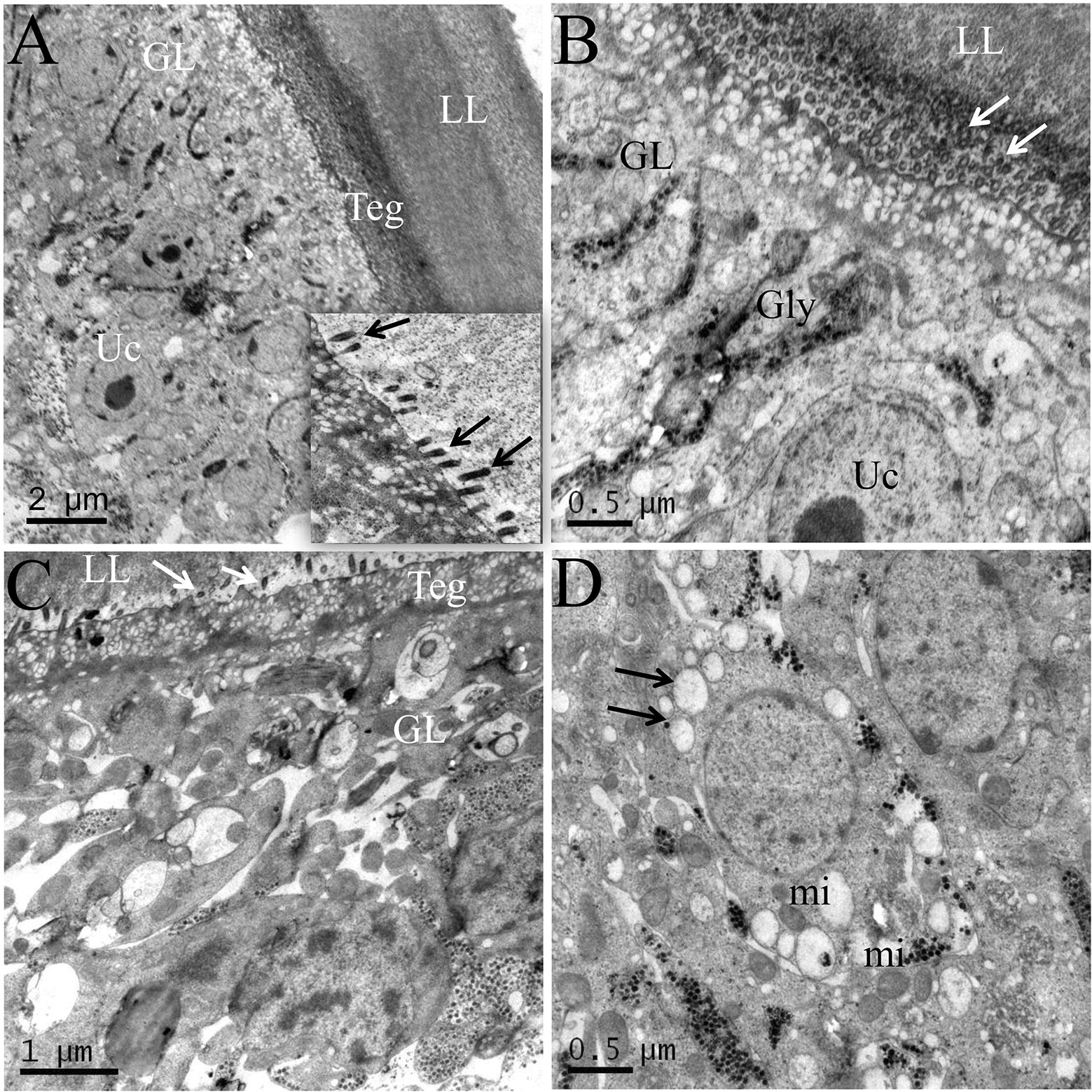
**TEM of** ***E. multilocularis*** **metacestodes incubated in vitro with 3-BrPA. A**, **B** The metacestodes of DMSO control show an intact morphology. LL: laminated layer, GL: germinal layer, Teg: tegument, Uc: undifferentiated cells, Gly: glycogen storage cells. The arrows indicate microtriches originating from the tegument and protruding into the germinated layer. **C**, **D** The metacestodes incubated with 40 μM 3-BrPA for 120 h show severe structural damages. The microtriches are shortened and reduced dramatically and even disappeared in most parts of the tegument. The germinal layer tissues show degeneration, largely filled with vacuoles (black arrows) and electron-dense mitochondria (mi).

On the basis of anti-*Echinococcus* effectivities of the selected glycolytic inhibitors, the effectivity of 3-BrPA, which exhibited the most active effect against *E. multilocularis*, was further confirmed on the ultrastructural level by SEM (Figure 3) and TEM (Figure 4). The *E. multilocularis* metacestodes vesicles in the DMSO control exhibited an intact typical structure: the outer acellular laminated layer attached closely with the tegument where many microtriches protruded distinctly into the laminated layer, and the germinal layer was surrounded by the laminated layer outside. The inner germinal layer was composed of glycogen storage cells and undifferentiated cells (Figures 3A, 4A, B). The metacestodes incubated with 40 μM 3-BrPA for 120 h showed noticeable damages: only residual cellular materials could be observed in many parts of the germinal layer tissues and in some regions, the germinal layers detached from the laminated layer (Figures 3B, C). After 120 h of incubation with 3-BrPA, the microtriches shortened and reduced dramatically, and even disappeared in most parts of the tegument (Figures 4C, D). The arrangement of the germinal layer became loose and largely filled with vacuoles. Furthermore, some regions of the germinal layer exhibited electron-dense mitochondria, suggesting that the parasites were metabolically impaired during the treatment (Figure 4D).

### Treatment of *E. multilocularis* infected mice with 3-BrPA

**Figure 5:**
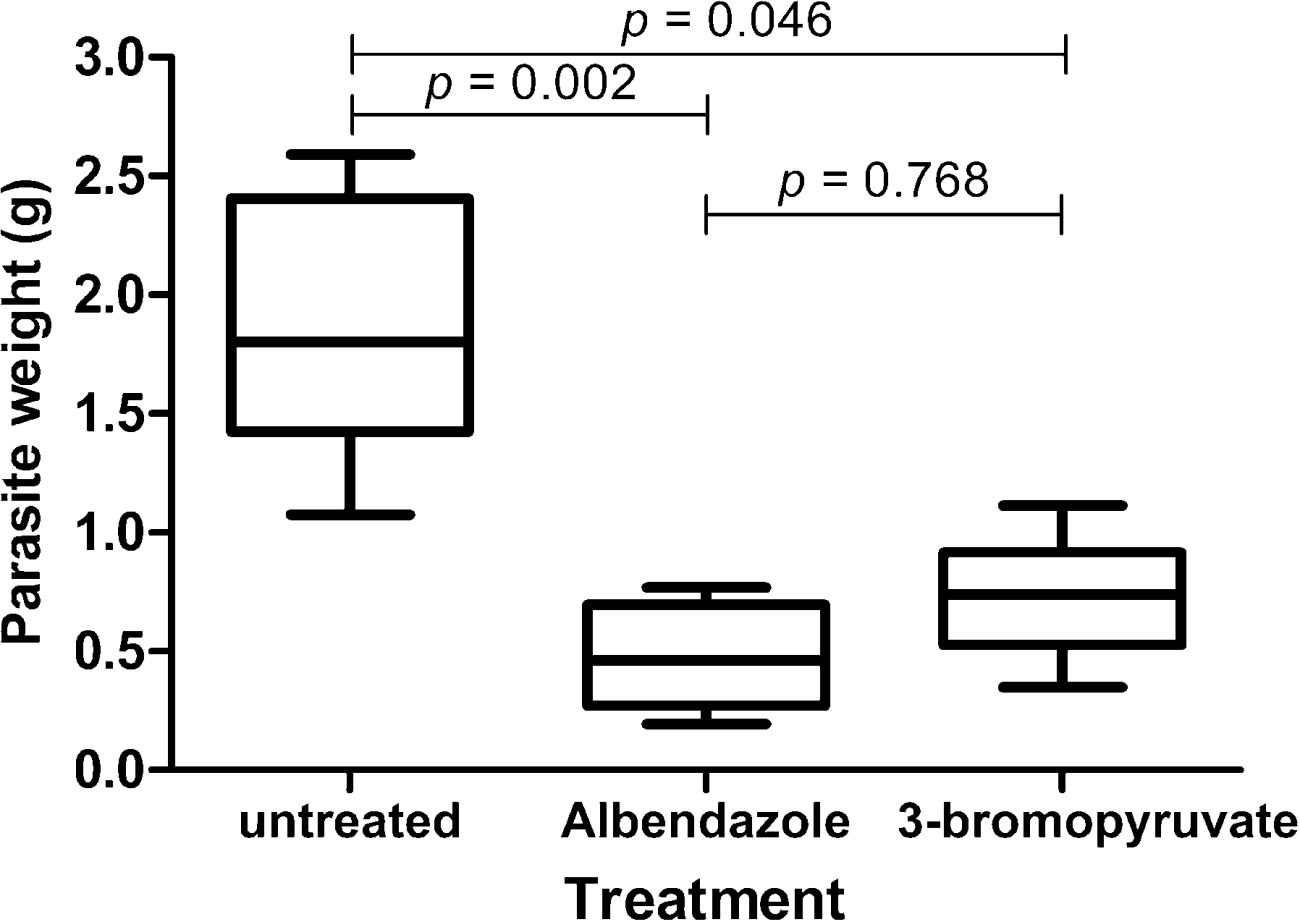
**Results of in vivo treatment of** ***E. multilocularis*-infected mice with 3-BrPA and ABZ.** The box plots indicate the distribution of parasite weights in different treatment groups. Significant reductions of parasite weights in relation to the untreated control group were achieved by treatment with 3-BrPA and ABZ. Although ABZ treatment was slightly more efficient than 3-BrPA, the difference did not show statistical significance (*p* = 0.768).

To investigate the in vivo therapeutic effect of 3-BrPA, BALB/c mice were intraperitoneally infected with *E. multilocularis* metacestodes and 8 weeks later administrated orally with ABZ (200 mg/kg/day) and 3-BrPA (25 mg/kg twice a week) respectively. After 6 weeks treatment, metacestode tissues were isolated from each of the experimental mice and the parasite weights were determined (Figure 5). Kruskal–Wallis analysis indicated a significant reduction of the wet weights of the metacestodes in both 3-BrPA (0.729 ± 0.256 g) and ABZ treated mice (0.477 ± 0.222 g) compared to those in the untreated group (1.860 ± 0.557 g). Although ABZ treatment seemed slightly more efficient than 3-BrPA, the difference in reduction of parasite weight did not show statistical significance (*p *= 0.768). Furthermore, no death and obvious adverse effects were observed in the treated mice.

### Cytotoxicity of 3-BrPA on human hepatocytes

**Figure 6:**
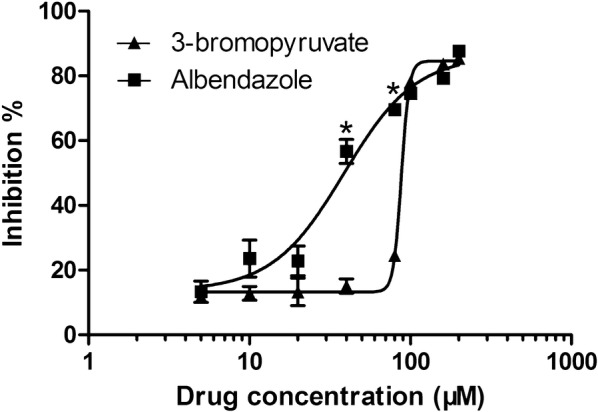
**Cytotoxicity of 3-BrPA and ABZ on human hepatocytes.** Cells were grown to confluence and incubated with 5, 10, 20, 40, 80, 100, 160 and 200 μM drugs for 48 h. Assessment was made by MTT reduction assay. Values are presented as percentages relative to the values for the untreated control group. *p < 0.05 vs. ABZ incubation.

The cytotoxicity of 3-BrPA and ABZ was assessed on human hepatocytes by MTT assay. In this assay, the cells were treated with a concentration series (5, 10, 20, 40, 80, 100, 160 and 200 μM) for 48 h (Figure 6). 3-BrPA and ABZ exhibited cytotoxicity in a concentration-dependent manner. At 5 μM, 11.6 ± 3.61% and 14.33 ± 8.15% human hepatocytes were inhibited by 3-BrPA and ABZ respectively. When the concentration increased to 200 μM, the inhibition of the hepatocyte viability reached 85.4 ± 1.06% by 3-BrPA and 87.7 ± 0.43% by ABZ. The IC_50_ value of 3-BrPA in human hepatocytes was 87.84 μM, which was higher than ABZ (38.7 μM). However, 3-BrPA at the effective dose against *E. granulosus* protoscoleces and *E. multilocularis* metacestodes did not show significant toxicity to human hepatocytes as ABZ did.

### Toxicology of 3-BrPA in mice

**Figure 7:**
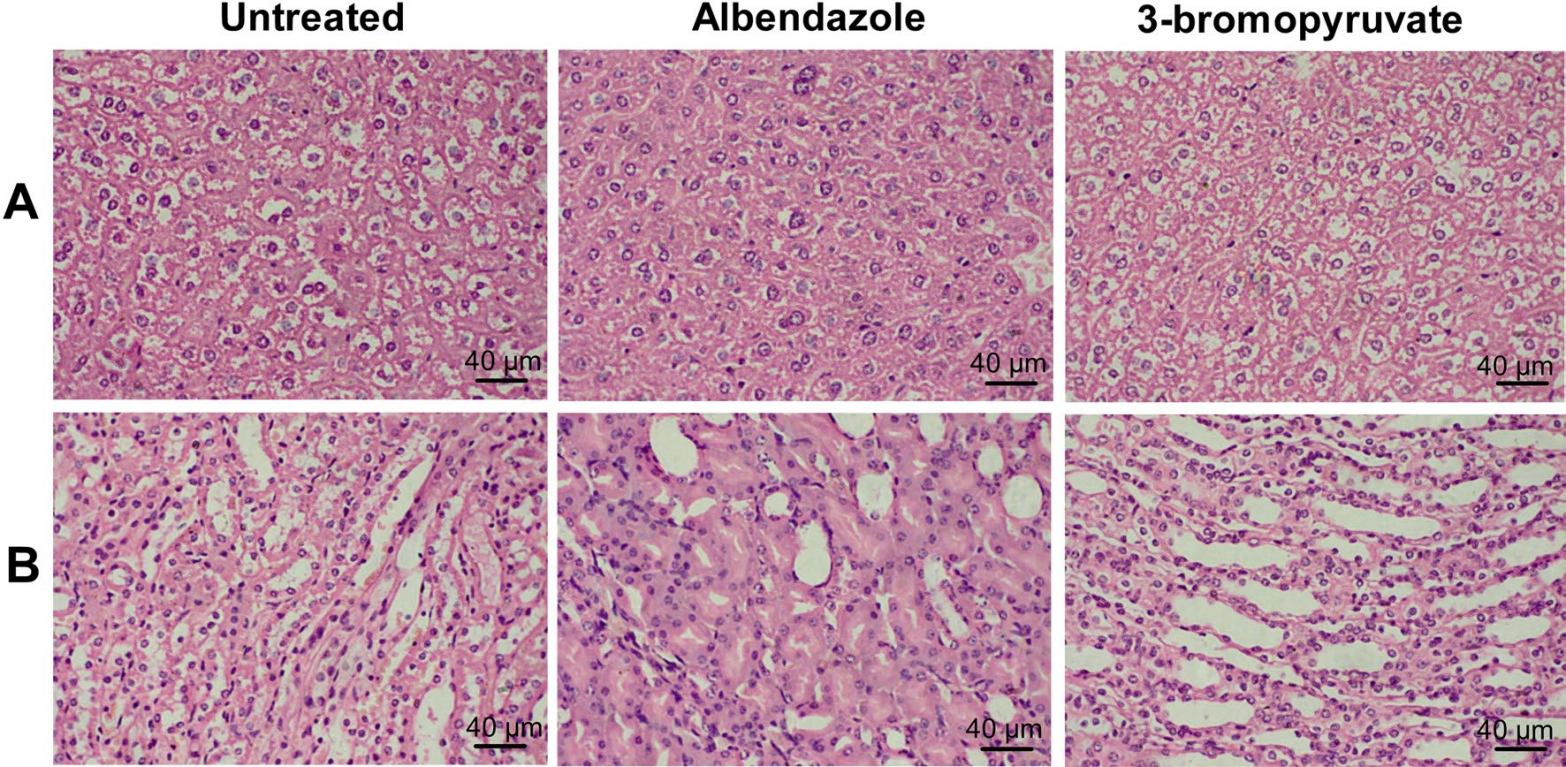
**Histopathological examination of liver and kidney in 3-BrPA treated mice.** When the mice were sacrificed, liver and kidney were collected and fixed in 10% of neutral formaldehyde and embedded in paraffin for HE staining. **A** liver; **B** kidney.

To investigate the in vivo toxicity of 3-BrPA, BALB/c mice were treated with 3-BrPA for 6 weeks. Table 1 presents the results of ALT, AST, TP, ALB, urea, CREA, ALP, GGT, TBIL, and DBIL levels in the serum of the mice after treatment. A significant increase (*p *= 0.033) in the level of AST was observed in the mice treated with ABZ compared with the untreated control group. In addition, ABZ treated mice showed a reduction of TP (*p *= 0.049) and ALB levels (*p *= 0.044). In contrast, no significant difference of the above biochemical analysis items was seen in 3-BrPA treated mice compared with the untreated control mice. These tests indicate no obvious effects on the liver and kidney function in the 3-BrPA treated mice. Histopathological examinations of the liver and the kidney in the treated mice confirmed the above results (Figure 7). The liver of ABZ treated mice exhibited distinct pathological changes: the spotty and focal necrosis, the hepatocyte edema, a number of inflammatory cells such as lymphocytes, the neutrophils infiltration, the dilation of partial portal area bile ducts, and the proliferation of fibrous connective tissue (Figure 7A). The histopathological examination in the kidney of ABZ treated mice revealed that the renal interstitium was hyperemic, and renal tubular epithelial cells swelled and had granular degeneration (Figure 7B) while the 3-BrPA treated mice did not show specific histopathological changes and injuries in either liver (Figure 7A) or kidney (Figure 7B).

**Table 1:** **Effect of 3-BrPA on biochemical parameters of BALB/c mice after 6** **weeks of oral administration**

Parameters	Untreated	ABZ treated	3-BrPA treated
TBIL (μM/L)	2.257 ± 0.232	2.071 ± 0.437	2.25 ± 0.206
DBIL (μM/L)	1.157 ± 0.396	1.000 ± 0.346	1.267 ± 0.359
IBIL (μM/L)	1.100 ± 0.227	1.071 ± 0.281	0.983 ± 0.380
TP (g/L)	57.786 ± 1.89	54.900 ± 2.196*	58.65 ± 1.447
ALB (g/L)	32.257 ± 1.011	30.942 ± 0.972*	32.667 ± 0.624
GLB (g/L)	25.529 ± 2.175	23.957 ± 1.987	25.983 ± 1.061
ALP (U/L)	92.571 ± 9.256	118.000 ± 45.997	95.167 ± 14.554
ALT (U/L)	26.833 ± 7.151	41.429 ± 19.617	35.717 ± 10.517
AST (U/L)	115.500 ± 18.355	148.000 ± 27.475*	129.167 ± 8.783
GGT (U/L)	1.543 ± 0.475	1.614 ± 0.706	1.267 ± 0.269
UREA (mM/L)	9.711 ± 1.249	9.154 ± 1.847	10.330 ± 1.492
CREA (μM/L)	27.886 ± 4.362	29.714 ± 3.862	28.050 ± 2.862

Data given as Mean ± SD of seven animals.

* Value significantly differs at 95% levels of significance (*p* < 0.05) for ABZ vs. untreated control.

## Discussion

In human or other intermediate hosts, the larval stage of *Echinococcus* depends on glycolysis for energy production and intermediates for other metabolic processes [22, 23] which implies that inhibition of glycolysis may preferentially eliminate the *Echinococcus* metacestodes. This is the first study examining the potential therapeutic implications of the glycolytic inhibitors on *E. granulosus* and *E. multilocularis*. In the present study, we investigated the in vitro and in vivo effects of several glycolytic inhibitors, such as 3-BrPA, ornidazole, clorsulon, sodium oxamate and NA-P_2_, against *E. granulosus* and *E. multilocularis*.

Ornidazole, as one of the nitroimidazole agents against anaerobes and protozoa, is used for the treatment of a variety of diseases, including intestinal amoebiasis, amoebic liver abscesses, anaerobic infections, giardiasis, and trichomoniasis [38–40]. Clorsulon is an aminobenzoldisulfonamide fasciolicide used in veterinary medicine [28]. CLS has been successfully applied in triclabendazole-resistant fascioliasis of sheep [41, 42]. It has been reported that the trematocidal effects of ornidazole and clorsulon depend on the inhibition of glycolytic enzymes phosphoglycerate kinase (PGK) and phosphoglyceratmutase (PGM) required by the trematodes for glycolysis [43, 44]. Oxamate is an established inhibitor of lactate dehydrogenase (LDH) via competition with pyruvate for the binding site on the enzyme. This compound has been recently proposed to be a promising anticancer drug that targets glycolysis [29–31]. 2,6-dihydroxynaphthalene (NA-P_2_) is an irreversible competitive inhibitor of hydroxynaphthaldehyde phosphate and exhibits a highly selective effect against the glycolytic enzyme fructose 1,6-bisphosphate aldolase (ALDO) from *Trypanosoma brucei* [32] and rabbit muscle [45]. 3-BrPA, a halogenated analog of pyruvic acid which exploits alkylating properties, is a potent inhibitor of hexokinase (HK) and glyceraldehyde 3-phosphate dehydrogenase (GAPDH) and effectively inhibits glycolysis [24–26]. It is effective against a wide variety of tumors in pre-clinical studies without apparent toxicity or recurrence [46] and shows promise in human cases [47], indicating that 3-BrPA is a fast, promising, specific, and effective anti-cancer agent. Moreover, 3-BrPA has also exhibited antiparasitic activity against *Trypanosoma brucei* [48], *Toxoplasma gondii* [49] and *Schistosoma mansoni* [50].

In our in vitro results, at an initial concentration of 40 μM, all of the selected glycolytic inhibitors showed considerable effects (the percentage of dead protoscoleces was over 65% after 7 days of treatment) against *E. granulosus* (Figure 1). In *E. multilocularis* metacestodes, each of the inhibitors could result in metacestode damages and loss of viability demonstrated by the increased *Em*AP activity in culture medium (Figure 2). According to our research results, the actions of these agents against both *E. granulosus* protoscoleces and *E. multilocularis* metacestodes are most probably exerted, as glycolytic inhibitors, by inhibiting the activities of glycolytic enzymes such as PGK, PGM, LDH, ALDO, HK and GAPDH. Among these inhibitors, 3-BrPA exhibited the highest activity against *Echinococcus*. In order to assess the anti-*Echinococcus* effect of 3-BrPA as a glycolysic inhibitor, ABZ and NTZ were used as positive controls. ABZ is one of the two drugs licensed for treatment of human Echinococcosis [11], and NTZ that exhibits a high parasitocidal activity in vitro against *Echinococcus* and therefore always serves as an efficient positive in vitro control to investigate the anti-*Echinococcus* activity of agents [51]. In the experiment, 3-BrPA exhibited a more efficacious effect than ABZ, and as efficacious as NTZ although their drug targets were completely different [52, 53].

The reason why 3-BrPA has a more active effect against *Echinococcus* than other selected glycolytic inhibitors might be due to the enzymes functioned by 3-BrPA. It has been documented that 3-BrPA prevents glucose from entering glycolysis by inhibiting HK [54]. As is well known, HK is the first one of a number of enzymes involved in glycolysis and more importantly, is a crucial rate-limiting enzyme that exerts main control on glycolytic flux. There are studies that have reported the inhibitory effects of 3-BrPA on *T. brucei* due to blockade of the GAPDH enzyme [55]. Thus, 3-BrPA may negatively affect the enzymatic function of GAPDH by pyruvylation and lead to *Echinococcus* death. Moreover, the 3-BrPA-mediated inhibition of HK can also impair the pentose phosphate pathway (PPP) and result in a reduction of ribose 5-phosphate synthesis that is crucial for parasite anabolism. At the same time, the inhibition of GAPDH can dampen the levels of its downstream-metabolite 3-phosphoglycerate and result in reductions of lipids and amino acids. Thus, it is indicated that the profound efficacy of 3-BrPA against *Echinococcus* may depend not only on the depletion of the ATP pool, but also the reduction of important intermediates of glycolysis for other metabolic processes. While other selected glycolytic inhibitors only dampen the downstream enzymes in the glycolysis pathway rather than HK.

The assay of concentration series of 3-BrPA demonstrated that the anti-*Echinococcus* effect of 3-BrPA was both dose- and time-dependent and was also highly active even at low concentrations. Moreover, like the parasitocidal agent NTZ, 3-BrPA exerted destructive effects rapidly on protoscoleces. Even at a lower concentration of 10 μM, it could kill 67% protoscoleces within 4 days. In addition, for treatment of metacestodes, 3-BrPA triggered a fast and dramatic increase in *Em*AP activity and distinct morphological alterations within 36 h compared with the ABZ treatment (Figure 2). This rapid action of 3-BrPA was also reported in *T. gondii* by de Lima et al. [49].

In the in vivo experiment, 3-BrPA-treated mice showed a statistically significant reduction in parasite weight compared to the untreated control, but not to ABZ treatment (*p *= 0.768). The reason for this might be the administration frequency of twice a week with 3-BrPA, which is much less than the daily treatment of ABZ. Although the treatment of 3-BrPA (25 mg/kg twice a week) exhibited a slightly less efficient activity than ABZ against metacestodes (in spite of no statistical difference), the in vivo toxicity assay showed almost no toxicity to the liver and kidney in the treated mice. In contrast, ABZ treated mice showed significant abnormalities in liver function, such as the reduction of TP and ALB levels and the increase in AST levels (Table 1 and Figure 7), accompanied with distinct pathological changes in the liver and kidney versus the untreated control, which was also consolidated by reports in human patients treated with ABZ as well [10, 11]. The levels of ALT, AST, TP, ALB, ALP, GGT, TBIL and DBIL are common markers for hepatic and biliary functionality. When the liver is damaged, the levels of serum ALT, AST, ALP, GGT, TBIL and DBIL will be increased, while those of TP and ALB will be decreased. Likewise, the serum levels of urea and CREA are common markers for kidney functions, and when glomerular filtration function is impaired, urea and CREA levels will be increased. Certainly, the significant difference of toxicity might be due to the same reason of the different administration frequency between these two drugs. However, if it is taken into consideration that 3-BrPA exhibited a higher IC_50_ value (87.84 μM) than ABZ (38.7 μM) in the in vitro cytotoxicity assay, and at the effective dose against *E. granulosus* protoscoleces and *E. multilocularis* metacestodes, 3-BrPA did not show significant toxicity to human hepatocytes, the conclusion can be easily interpreted. In addition, our result is also supported by a study in mouse hepatocellular carcinoma which highlights the ability of 3-BrPA to eradicate cancer cells without causing significant toxicity to normal liver tissue or recurrence [56]. As is well known that in 70% of CE patients and in nearly 100% of AE patients, the parasite lesion occurs in the liver, leading to severe impairment of the liver. Therefore, in comparison to ABZ, the nearly non-toxic property of 3-BrPA treatment to the liver indicates that 3-BrPA might be a promising alternative to ABZ in the treatment of echinococcosis.

In conclusion, in the present study, we demonstrate for the first time that the glycolytic inhibitors exhibit effective in vitro activity against both *E. granulosus* and *E. multilocularis*. The in vivo results of 3-BrPA also consolidate the anti-AE effect. Hence, implication of the glycolytic inhibitors might be a novel chemotherapeutical option of echinococcosis and glycolytic enzymes might be valuable and promising drug targets in *Echinococcus*. Furthermore, 3-BrPA, exhibiting a profound activity against *Echinococcus* and no host toxicity both in vitro and in vivo, will be a promising drug for the treatment of echinococcosis and worthy of further investigation.

## References

[1] Cadavid Restrepo AM, Yang YR, McManus DP, Gray DJ, Giraudoux P, Barnes TS, Williams GM, Soares Magalhães RJ, Hamm NA, Clements AC (2016). The landscape epidemiology of echinococcoses. Infect Dis Poverty.

[2] Chai JJ (2009). Echinococcosis control in China: challenges and research needs. Zhongguo Ji Sheng Chong Xue Yu Ji Sheng Chong Bing Za Zhi.

[3] Wang LY, Wu WP, Zhu XH (2010). The endemic status of hydatidosis in China from 2004 to 2008. Chin J Zoonoses.

[4] Eckert J, Deplazes P (2004). Biological, epidemiological, and clinical aspects of echinococcosis, a zoonosis of increasing concern. Clin Microbiol Rev.

[5] Torgerson PR, Keller K, Magnotta M, Ragland N (2010). The global burden of alveolar echinococcosis. PLoS Negl Trop Dis.

[6] Torgerson PR, Macpherson CN (2011). The socioeconomic burden of parasitic zoonoses: global trends. Vet Parasitol.

[7] Brunetti E, Kern P, Vuitton DA, Writing Panel for the WHO-IWGE (2010). Expert consensus for the diagnosis and treatment of cystic and alveolar echinococcosis in humans. Acta Trop.

[8] Vuitton DA (2009). Benzimidazoles for the treatment of cystic and alveolar echinococcosis: what is the consensus?. Expert Rev Anti Infect Ther.

[9] Reuter S, Jensen B, Buttenschoen K, Kratzer W, Kern P (2000). Benzimidazoles in the treatment of alveolar echinococcosis: a comparative study and review of the literature. J Antimicrob Chemother.

[10] Stojkovic M, Zwahlen M, Teggi A, Vutova K, Cretu CM, Virdone R, Nicolaidou P, Cobanoglu N, Junghanss T (2009). Treatment response of cystic echinococcosis to benzimidazoles: a systematic review. PLoS Negl Trop Dis.

[11] Davis A, Pawlowski ZS, Dixon H (1986). Multicentre clinical trials of benzimidazolecarbamates in human echinococcosis. Bull World Health Organ.

[12] Spicher M, Naguleswaran A, Ortega-Mora LM, Müller J, Gottstein B, Hemphill A (2008). In vitro and in vivo effects of 2-methoxyestradiol, either alone or combined with albendazole, against *Echinococcus* metacestodes. Exp Parasitol.

[13] Hemer S, Brehm K (2012). In vitro efficacy of the anticancer drug imatinib on *Echinococcus multilocularis* larvae. Int J Antimicrob Agents.

[14] Liance M, Nemati F, Bories C, Couvreur P (1993). Experience with doxorubicin-bound polyisohexylcyanoacrylate nanoparticles on murine alveolar echinococcosis of the liver. Int J Parasitol.

[15] Liance M, Bresson-Hadni S, Vuitton DA, Lenys D, Carbillet JP, Houin R (1992). Effects of cyclosporin A on the course of murine alveolar echinococcosis and on specific cellular and humoral immune responses against *Echinococcus multilocularis*. Int J Parasitol.

[16] Naguleswaran A, Spicher M, Vonlaufen N, Ortega-Mora LM, Torgerson P, Gottstein B, Hemphill A (2006). In vitro metacestodicidal activities of genistein and other isoflavones against *Echinococcus multilocularis* and *Echinococcus granulosus*. Antimicrob Agents Chemother.

[17] Spicher M, Roethlisberger C, Lany C, Stadelmann B, Keiser J, Ortega-Mora LM, Gottstein B, Hemphill A (2008). In vitro and in vivo treatments of echinococcus protoscoleces and metacestodes with artemisinin and artemisinin derivatives. Antimicrob Agents Chemother.

[18] Nicolao MC, Elissondo MC, Denegri GM, Goya AB, Cumino AC (2014). In vitro and in vivo effects of tamoxifen against larval stage *Echinococcus granulosus*. Antimicrob Agents Chemother.

[19] Stadelmann B, Aeschbacher D, Huber C, Spiliotis M, Müller J, Hemphill A (2014). Profound activity of the anti-cancer drug bortezomib against *Echinococcus multilocularis* metacestodes identifies the proteasome as a novel drug target for cestodes. PLoS Negl Trop Dis.

[20] Pensel PE, Albani C, Gamboa GU, Benoit JP, Elissondo MC (2014). In vitro effect of 5-fluorouracil and paclitaxel on *Echinococcus granulosus* larvae and cells. Acta Trop.

[21] Pensel PE, Elissondo N, Gambino G, Gamboa GU, Benoit JP, Elissondo MC (2017). Experimental cystic echinococcosis therapy: in vitro and in vivo combined 5-fluorouracil/albendazole treatment. Vet Parasitol.

[22] Agosin M (1957). Studies on the metabolism of *Echinococcus* *granulosus*. II. Some observations on the carbohydrate metabolism of hydatid cyst scolices. Exp Parasitol.

[23] McManus DP, Smyth JD (1978). Differences in the chemical composition and carbohydrate metabolism of *Echinococcus granulosus* (horse and sheep strains) and *E. multilocularis*. Parasitology.

[24] Ko YH, Pedersen PL, Geschwind JF (2001). Glucose catabolism in the rabbit VX2 tumor model for liver cancer: characterization and targeting hexokinase. Cancer Lett.

[25] Zhang Q, Zhang Y, Zhang P, Chao Z, Xia F, Jiang C, Zhang X, Jiang Z, Liu H (2014). Hexokinase II Inhibitor, 3-BrPA induced autophagy by stimulating ROS formation in human breast cancer cells. Genes Cancer.

[26] Xu RH, Pelicano H, Zhou Y, Carew JS, Feng L, Bhalla KN, Keating MJ, Huang P (2005). Inhibition of glycolysis in cancer cells: a novel strategy to overcome drug resistance associated with mitochondrial respiratory defect and hypoxia. Cancer Res.

[27] Lamp KC, Freeman CD, Klutman NE, Lacy MK (1999). Pharmacokinetics and pharmacodynamics of the nitroimidazole antimicrobials. Clin Pharmacokinet.

[28] Elitok B, Elitok OM, Kabu M (2006). Field trial on comparative efficacy of four fasciolicides against natural liver fluke infection in cattle. Vet Parasitol.

[29] Thornburg JM, Nelson KK, Clem BF, Lane AN, Arumugam S, Simmons A, Eaton JW, Telang S, Chesney J (2008). Targeting aspartate aminotransferase in breast cancer. Breast Cancer Res.

[30] Fiume L, Vettraino M, Manerba M, Di Stefano G (2011). Inhibition of lactic dehydrogenase as a way to increase the anti-proliferative effect of multi-targeted kinase inhibitors. Pharmacol Res.

[31] Fujiwara S, Kawano Y, Yuki H, Okuno Y, Nosaka K, Mitsuya H, Hata H (2013). PDK1 inhibition is a novel therapeutic target in multiple myeloma. Br J Cancer.

[32] Dax C, Duffieux F, Chabot N, Coincon M, Sygusch J, Michels PA, Blonski C (2006). Selective irreversible inhibition of fructose 1,6-bisphosphate aldolase from *Trypanosoma brucei*. J Med Chem.

[33] Walker M, Rossignol JF, Torgerson P, Hemphill A (2004). In vitro effects of nitazoxanide on *Echinococcus granulosus* protoscoleces and metacestodes. J Antimicrob Chemother.

[34] Reuter S, Merkle M, Brehm K, Kern P, Manfras B (2003). Effect of amphotericin B on larval growth of *Echinococcus multilocularis*. Antimicrob Agents Chemother.

[35] Stettler M, Siles-Lucas M, Sarciron E, Lawton P, Gottstein B, Hemphill A (2001). *Echinococcus multilocularis* alkaline phosphatase as a marker for metacestode damage induced by in vitro drug treatment with albendazole sulfoxide and albendazole sulfone. Antimicrob Agents Chemother.

[36] Tada H, Shiho O, Kuroshima K, Koyama M, Tsukamoto K (1986). An improved colorimetric assay for interleukin 2. J Immunol Methods.

[37] Serafini S, Santos MM, Aoun Tannuri AC, Zerbini MCN, de Mendonça Coelho MC, de Oliveira Gonçalves J, Tannuri U (2017). Is hematoxylin–eosin staining in rectal mucosal and submucosal biopsies still useful for the diagnosis of Hirschsprung disease?. Diagn Pathol.

[38] Jokipii L, Jokipii AM (1982). Treatment of giardiasis: comparative evaluation of ornidazole and tinidazole as a single oral dose. Gastroenterology.

[39] Martin C, Bruguerolle B, Mallet MN, Condomines M, Sastre B, Gouin F (1990). Pharmacokinetics and tissue penetration of a single dose of ornidazole (1,000 milligrams intravenously) for antibiotic prophylaxis in colorectal surgery. Antimicrob Agents Chemother.

[40] Rossignol JF, Maisonneuve H, Cho YW (1984). Nitroimidazoles in the treatment of trichomoniasis, giardiasis, and amebiasis. Int J Clin Pharmacol Ther Toxicol.

[41] Coles GC, Stafford KA (2001). Activity of oxyclozanide, nitroxynil, clorsulon and albendazole against adult triclabendazole-resistant *Fasciola hepatica*. Vet Rec.

[42] Hutchinson GW, Dawson K, Fitzgibbon CC, Martin PJ (2009). Efficacy of an injectable combination anthelmintic (nitroxynil + clorsulon + nitroxynil + clorsulon + ivermectin) against early immature *Fasciola hepatica* compared to triclabendazole combination flukicides given orally or topically to cattle. Vet Parasitol.

[43] Schulman MD, Ostlind DA, Valentino D (1982). Mechanism of action of MK-401 against *Fasciola hepatica*: inhibition of phosphoglycerate kinase. Mol Biochem Parasitol.

[44] Jones AR, Cooper TG (1997). Metabolism of 36Cl-ornidazole after oral application to the male rat in relation to its antifertility activity. Xenobiotica.

[45] Dax C, Coinçon M, Sygusch J, Blonski C (2005). Hydroxynaphthaldehyde phosphate derivatives as potent covalent Schiff base inhibitors of fructose-1,6-bisphosphate aldolase. Biochemistry.

[46] Ganapathy-Kanniappan S, Vali M, Kunjithapatham R, Buijs M, Syed LH, Rao PP, Ota S, Kwak BK, Loffroy R, Geschwind JF (2010). 3-bromopyruvate: a new targeted antiglycolytic agent and a promise for cancer therapy. Curr Pharm Biotechnol.

[47] Ko YH, Verhoeven HA, Lee MJ, Corbin DJ, Vogl TJ, Pedersen PL (2012). A translational study “case report” on the small molecule “energy blocker” 3-bromopyruvate (3BP) as a potent anticancer agent: from bench side to bedside. J Bioenerg Biomembr.

[48] Vanderheyden N, Wong J, Docampo R (2000). A pyruvate-proton symport and an H + -ATPase regulate the intracellular pH of *Trypanosoma brucei* at different stages of its life cycle. Biochem J.

[49] de Lima LP, Seabra SH, Carneiro H, Barbosa HS (2015). Effect of 3-bromopyruvate and atovaquone on infection during in vitro interaction of *Toxoplasma gondii* and LLC-MK2 cells. Antimicrob Agents Chemother.

[50] Manneck T, Keiser J, Müller J (2012). Mefloquine interferes with glycolysis in schistosomula of *Schistosoma mansoni* via inhibition of enolase. Parasitology.

[51] Stettler M, Fink R, Walker M, Gottstein B, Geary TG, Rossignol JF, Hemphill A (2003). In vitro parasiticidal effect of nitazoxanide against *Echinococcus multilocularis* metacestodes. Antimicrob Agents Chemother.

[52] Lacey E (1988). The role of the cytoskeletal protein, tubulin, in the mode of action and mechanism of drug resistance to benzimidazoles. Int J Parasitol.

[53] Sisson G, Goodwin A, Raudonikiene A, Hughes NJ, Mukhopadhyay AK, Berg DE, Hoffman PS (2002). Enzymes associated with reductive activation and action of nitazoxanide, nitrofurans, and metronidazole in *Helicobacter pylori*. Antimicrob Agents Chemother.

[54] Chen Z, Zhang H, Lu W, Huang P (2009). Role of mitochondria associated hexokinase II in cancer cell death induced by 3-bromopyruvate. Biochim Biophys Acta.

[55] Barnard JP, Reynafarje B, Pedersen PL (1993). Glucose catabolism in African trypanosomes. Evidence that the terminal step is catalyzed by a pyruvate transporter capable of facilitating uptake of toxic analogs. J Biol Chem.

[56] Kim W, Yoon JH, Jeong JM, Cheon GJ, Lee TS, Yang JI, Park SC, Lee HS (2007). Apoptosis-inducing antitumor efficacy of hexokinase II inhibitor in hepatocellular carcinoma. Mol Cancer Ther.

